# Emergency Carotid Endarterectomy Instead of Carotid Artery Stenting Reduces Delayed Hemorrhage in Thrombectomy Stroke Patients

**DOI:** 10.1007/s00062-020-00954-7

**Published:** 2020-09-17

**Authors:** Raveena Singh, Sven Dekeyzer, Arno Reich, Drosos Kotelis, Alexander Gombert, Martin Wiesmann, Omid Nikoubashman

**Affiliations:** 1 Department of Neuroradiology, University Hospital RWTH Aachen University, Pauwelsstr. 30, 52074 Aachen, Germany; 2 Department of Neurology, University Hospital RWTH Aachen University, Pauwelsstr. 30, 52074 Aachen, Germany; 3 Department of Vascular Surgery, University Hospital RWTH Aachen University, Pauwelsstr. 30, 52074 Aachen, Germany

**Keywords:** Stroke, Thrombectomy, Carotid endarterectomy, Carotid stenting, Hemorrhage

## Abstract

**Purpose:**

Data in the literature suggest that thrombectomy with emergency carotid artery stenting (CAS) in acute stroke is associated with an increased hemorrhage rate. As we perform thrombectomy with the patient under general anesthesia, we avoid emergency CAS and perform emergency carotid endarterectomy (CEA) as an alternative to CAS in the same anesthesia session in our angiography suite whenever needed and possible.

**Methods:**

We compared 27 thrombectomy patients with emergency CEA and 62 thrombectomy patients with emergency CAS and glycoprotein (Gp) IIb/IIIa inhibitors and/or dual antiplatelet therapy (DAPT) in the same time span.

**Results:**

The symptomatic hemorrhage rate was 0% (0/27) in the CEA group and 8% (5/62) in the CAS group (*p* = 0.317). The parenchymal hemorrhage rate (PH2) was 7% (2/27) in the CEA group and 16% (10/62) in the CAS group (*p* = 0.333). Both cases of PH2 in the CEA group occurred during the intervention and were diagnosed on immediate postinterventional imaging, whereas in the CAS group only 2/10 cases of PH2 occurred during the intervention and the remaining 8 PH2 occurred within 3 days after the intervention (*p* = 0.048). Clinical outcome at 90 days was comparable with 39% of CEA and 51% of CAS patients achieving good clinical outcome (modified Rankin scale, mRS 0–2, *p* = 0.452).

**Conclusion:**

The use of CEA is a feasible alternative to CAS in acute stroke and has the advantage that DAPT/GpIIb/IIIa inhibitors are not needed. All PH2 in CEA patients occurred during the intervention, implying that hemorrhage in this group is likely to be caused by reperfusion injury, whereas delayed hemorrhage is likely to be caused by DAPT/GpIIb/IIIa inhibitors.

## Introduction

Stroke is one of the most common causes of disability and death worldwide. Endovascular stroke treatment allows for treatment of patients with large vessel occlusion stroke [1, 2]. Approximately 20% of ischemic strokes are due to atherosclerotic carotid artery stenosis and approximately 6% of patients who receive endovascular stroke treatment need additional emergency carotid artery stenting (CAS) for sufficient stroke therapy [3, 4]. It has been shown that compared to thrombolysis alone, patients with additional CAS benefit from endovascular treatment despite the inherent risks of the procedure [5]; however, CAS and subsequent (obligatory) dual antiplatelet therapy (DAPT) appear to be associated with an increased risk for parenchymal hemorrhage (PH) of up to 43% [6–10].

The CAS procedure in thrombectomy patients is usually performed either before thrombectomy to gain access to the occlusion site, or after thrombectomy to treat a high-grade stenosis. If treatment of a carotid stenosis is not urgent, it is common practice in many hospitals to postpone the treatment of the carotid artery and to perform either CAS or carotid endarterectomy (CEA) in the subacute phase after thrombectomy. Recently, Slawski et al. reported on 12 cases in which emergency CEA was performed after thrombectomy [11]. The authors reported that symptomatic intracerebral hemorrhage (sICH) occurred significantly less frequently in CEA patients compared to CAS patients; however, the practice of their emergency CEA is not further specified as the exact timepoint of the procedure and patient management in terms of logistics and anesthesia were unclear. For our study, we analyzed thrombectomy patients who received either CEA or CAS in the same anesthesia session as the thrombectomy and focused our analysis on the PH rate, which is one of the most relevant factors for clinical decision making after stroke (e.g. modification of antiplatelet medication or surgery). Considering that there is no need for DAPT when CEA is performed instead of CAS, we hypothesized that our procedure would result in lower PH rates and improved clinical outcome.

## Patients and Methods

Data for this study were based on our prospectively maintained stroke registry. This retrospective analysis was approved by our local ethics board, allowing us to use all patient data without explicit consent.

Our hospital as a tertiary stroke center has a coverage of approximately 1.2 million inhabitants. Patient selection and procedures have been reported previously [12, 13]. For thrombectomy all commonly used techniques are performed in our clinic, such as stent-retriever thrombectomy, ADAPT (a direct aspiration first pass technique) and combined approaches, such as SAVE (stent-retriever assisted vacuum-locked extraction) and Solumbra; for carotid stenting the standard device is the Wallstent (Boston Scientific, Malborough, MA, USA). To avoid CAS and DAPT/GpIIb/IIIa inhibitors we established an interdisciplinary consensus in our hospital to perform thrombectomy with general anesthesia and CEA in the same anesthesia session in our angiography suite either before or after thrombectomy. We can hereby eliminate the need for a new general anesthesia and avoid blood pressure drops that may cause further tissue injury through ischemia. Our rationale is to avoid acute treatment of ICA stenosis altogether whenever possible, but to perform CEA 1) before or 2) after thrombectomy whenever necessary. We notify our vascular surgery team, which is available 24/7, whenever 1) surgical access to the occlusion site is needed because access to the occlusion site via femoral or radial/brachial access is expected to be difficult and to take longer than 45 min or 2) CAS and DAPT after the procedure are to be avoided (e.g., if the hemorrhage risk is increased because the patient is anticoagulated or the infarction volume is large). The vascular surgery team is then on hold in our angiography suite and performs the necessary procedure in the same anesthesia session before or after thrombectomy. No modifications of pharmaceutical treatment are made for surgery; hence, intravenous thrombolysis is continued during surgery. If not otherwise contraindicated, all of our CAS patients receive GpIIb/IIIa inhibitors during and DAPT after intervention as suggested by the European Recommendations on Organization of Interventional Care in Acute Stroke (EROICAS) to avoid thromboembolism [14]. One patient in the CAS group, who eventually suffered from hemorrhage, did not receive a GpIIb/IIIa inhibitor during the intervention, for which we were unable to specify the motive retrospectively.

Between June 2013 and September 2018, a total of 114 consecutive patients in our clinic with occlusions of the anterior circulation received thrombectomy together with either emergency CAS or CEA. To homogenize our patient population, we excluded 21 patients with carotid dissection and 2 patients were excluded from further analysis as they received emergency stenting as well as emergency CEA during hospitalization. One further patient was excluded from analysis because there was a hemorrhage prior to thrombectomy and another one was excluded because imaging data regarding hemorrhage was not adequate. This left 89 patients to be included in our study (27 CEA and 62 CAS patients).

Primary outcome parameters were the occurrence of space-occupying parenchymal hemorrhage (defined as PH1 and PH2 according to the European Australasian Acute Stroke Study, ECASS, with PH1 defined as a hematoma <30% of the infarcted area with mild space-occupying effect and PH2 defined as a hematoma >30% of the infarcted area with significant space-occupying effect) [15, 16]. Secondary outcome measures were the occurrence of sICH (parenchymal hemorrhage accompanied by neurological deterioration of ≥4 points on the National Institutes of Health Stroke Scale, NIHSS), final infarction size according to the Alberta Stroke Program Early CT Score (ASPECTS), the rate of good clinical outcome according to the modified Rankin scale (mRS 0–2) and mortality, both after 90 days [17–19]. We classified PH into a) intrainterventional hemorrhages, if they were seen on immediate postinterventional imaging, which all patients received and b) delayed hemorrhages if they were diagnosed on the obligatory imaging 24 h after the procedure or any further postinterventional imaging indicated by clinical deterioration. Clinical, procedural, and imaging data included among others baseline characteristics (such as age, sex, and initial stroke size) and an extended set of variables, such as arterial pressure during the procedure (Table 1). As our patient cohort was heterogeneous with patients having received CAS/CEA before and after thrombectomy, puncture to revascularization times are not an adequate measure for the procedural length. Because all patients in our institution undergo computed tomography (CT) imaging immediately after the procedure, we indicate puncture to postinterventional CT as a procedural marker that includes all treatment-associated times.

**Table 1:** Baseline characteristics, procedural data, and outcome parameters

	CEA group (*n* = 27)	CAS group (*n* = 62)	p*
**Baseline characteristics and procedural data**			
Age (years, median)	73 (IQR 62–82)	71 (IQR 63–78)	0.492
Female sex (*n*)	12/27 (44%)	17/62 (27%)	0.143
NIHSS^a^ upon admission (*n*)	18 (IQR 12–21)	15 (IQR 9–17)	0.052
ASPECTS^b^ upon admission (*n*)	9 (IQR 7–9)	9 (IQR 7–10)	0.793
Intravenous (IV) thrombolysis (*n*)	13/27 (48%)	39/62 (63%)	0.244
Intraarterial (IA) thrombolysis (*n*)	0/27	2/62 (3%)	1.00
IV + IA thrombolysis (*n*)	3/27 (11%)	1/62 (2%)	0.081
No thrombolysis (*n*)	11/27 (41%)	20/62 (32%)	0.475
Maximum systolic blood pressure during anesthesia (mm Hg) (median)	170 ± 18	165 ± 22	0.072
Procedural timespan: puncture to postinterventional CT (min)	250 ± 70	172 ± 49	<0.001
*Medical history*			
Hypertension (*n*)	25/27(93%)	51/62 (82%)	0.329
Diabetes mellitus (*n*)	8/27 (30%)	14/62 (23%)	0.594
Active smoker (*n*)	10/27 (37%)	24/61 (39%)	1.0
Previous stroke (*n*)	5/27 (19%)	9/62 (15%)	0.753
*Previous medication*			
Aspirin (ASA) (*n*)	6/27 (22%)	20/62 (32%)	0.449
Clopidogrel (CPG) (*n*)	2/27 (7%)	0/62	0.090
Oral anticoagulants (OAC) (*n*)	0/27	1/62 (2%)	1.00
ASA + CPG (*n*)	0/27	2/62 (3%)	1.00
ASA + OAC (*n*)	2 (7%)	0/62	0.090
CPG + OAC (*n*)	0/27	0/62	–
ASA + CPG + OAC (*n*)	0/27	1/62 (2%)	1.00
No previous medication (*n*)	17/27 (63%)	38/62 (61%)	1.00
**Hemorrhage (primary outcome)**			
PH1^c^ (intrainterventional) (*n*)	1/27(4%)	0	0.303
PH2^c^ (intrainterventional) (*n*)	2/27(7%)	2/62 (3%)	0.582
PH1 and PH2 (intrainterventional) (*n*)	3/27(11%)	2/62 (3%)	0.161
PH1 (postinterventional) (*n*)	1/27(4%)	2/62 (3%)	1
PH2 (postinterventional) (*n*)	0	8/62 (13%)	0.048
PH1 and PH2 (postinterventional) (*n*)	1/27(4%)	10/62 (16%)	0.162
PH1 (regardless of timing) (*n*)	2/27(7%)	2/62 (3%)	0.582
PH2 (regardless of timing) (*n*)	2/27(7%)	10/62 (16%)	0.333
PH1 and PH2 (regardless of timing) (*n*)	4/27 (15%)	12/62 (19%)	0.768
**Secondary outcome parameters**			
sICH^d^ (intra-interventional) (*n*)	0	1/62 (2%)	1
sICH (post-interventional) (*n*)	0	4/62 (7%)	0.310
sICH (regardless of timing) (*n*)	0	5/62 (8%)	0.317
Lethal hemorrhage (intra-interventional) (*n*)	0	0	0
Lethal hemorrhage (post-interventional) (*n*)	0	2/62 (3%)	1
Final infarction size (ASPECTS) (median)	6 (IQR 5–9)	7 (IQR 5–8)	0.996
mRS^e^ 0–2 at 3 months (*n*)	9/23 (39*%)*	26/51 (51%)	0.452
Death at 3 months (*n*)	8/23 (35%)	11/51 (22%)	0.259

*ASA* aspirin, *CAE* carotid endarterectomy, *CAS* carotid artery stenting, *CPG* clopidogrel, *CT* computed tomography, *IA* intraarterial, *IV* intraavenous, *OAC* oral anticoagulants

^a^National Institutes of Health Stroke Scale

^b^Alberta Stroke Program Early CT Score

^c^Parenchymal hemorrhage type 1 and 2

^d^Symptomatic intracranial hemorrhage

^e^Modified Rankin Scale

### Statistics

Parametric variables are indicated as mean ± standard deviation (SD) and non-parametric variables are indicated as median and interquartile range (IQR). For comparison of these variables we used a Student’s t‑test or a Mann–Whitney *U-*test after testing for normal distribution with a Shapiro-Wilk test. Nominal variables were tested with one-sided and two-sided Fisher’s exact tests and Χ^2^ tests, depending on the prior hypothesis and sample size. *P-*values under the α‑level of 0.05 were defined as significant. Multivariable analysis was performed with a binary logistic regression test indicating odds ratios (OR) and 95% confidence intervals (CI). All statistical analyses were performed with IBM SPSS Statistics V25.0 (IBM, Armonk, NY, USA) software.

## Results

**Fig. 1:**
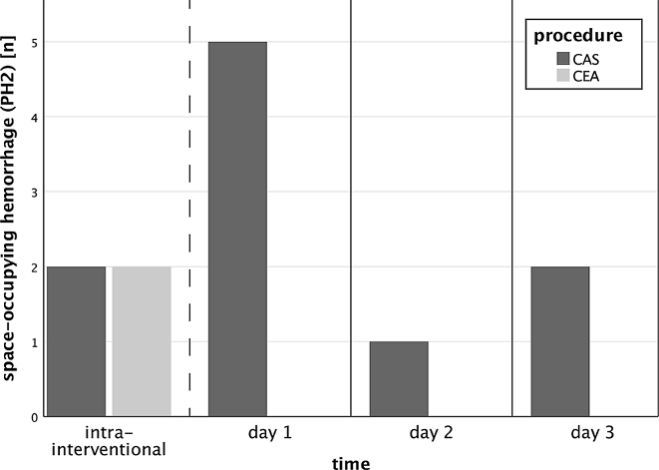
Occurrence of space-occupying hemorrhage (PH2) in thrombectomy patients depending on the procedure. *CAS* carotid artery stenting (62 patients: 2 intrainterventional PH2, 5 PH2 on day 1, 1 PH2 on day 2, 2 PH2 on day 3 after the intervention), *CEA* carotid endarterectomy (27 patients: 2 intrainterventional PH2, there was no delayed PH2 in the CEA group)

Baseline and outcome parameters of CAS and CEA patients including age, sex, NIHSS, ASPECTS, rate of IV and IA thrombolysis, medical history, previous medication and maximum systolic blood pressure during the intervention were comparable and are summarized in Table 1 (*p* ≥ 0.052). The NIHSS upon admission of patients who eventually received CEA was not significantly worse than in those who received CAS (18, IQR 12–21 vs. 15, IQR 9–17; *p* = 0.052). The CEA patients also tended to have a non-significantly higher incidence for comorbidities, such as arterial hypertension (93% vs. 82%), diabetes mellitus (30% vs. 23%) and previous stroke (19% vs. 15%). In summary, the CAS group suffered twice as often from PH2 (16% vs. 7%), albeit no statistical significance was reached (Table 1). All space-occupying hemorrhages (PH2) occurred within 3 days after interventions (Fig. 1). More specifically, both PH2 in the CEA group occurred during the intervention and were diagnosed on immediate postinterventional imaging with no delayed PH2. On the other hand, 2/10 PH2 in the CAS group occurred during the intervention and the remaining 8 PH2 occurred within 3 days after the intervention (*p* = 0.048). Logistic regression multivariable analysis revealed that the risk for delayed PH2 to occur after CEA is less than after CAS (*p* = 0.048, OR 0.871; CI 0.791–0.959). Regarding all parenchymal hematomas (PH1 and PH2), all 12 cases in the CAS group occurred while the patients were under DAPT (*n* = 10) or GpIIb/IIIa inhibitor therapy (*n* = 2). None of the CEA patients with hemorrhages were under platelet inhibition when the hemorrhage occurred. Maximum systolic blood pressure between patients with and without intrainterventional hemorrhage (PH2) did not differ significantly (155 ± 26 vs. 165 ± 21 mm Hg; *p* = 0.317). The procedural duration (puncture to postinterventional CT) in CEA patients was significantly longer than in CAS patients (250 ± 70 min vs. 172 ± 49 min; *p* < 0.001). Clinical outcome at 90 days did not differ significantly between the groups, with 39% of CEA and 51% of CAS patients achieving good clinical outcome (mRS 0–2, *p* = 0.452).

## Discussion

Our study showed that CEA in thrombectomy patients performed in the angiography suite in the same anesthesia session is a feasible and safe procedure, with no surgery-related complications in 27 CEA patients despite hemorrhagic risk factors, such as intravenous thrombolysis. More importantly, the finding of our study that timing of the hemorrhage differs significantly allows interesting insights into the pathomechanisms of hemorrhage in CEA and CAS patients. We found that intrainterventional hemorrhage rates were comparable, but that delayed hemorrhage was significantly more frequent in the CAS group.

The fact that the rate of large parenchymal hemorrhages (PH2) on immediate postinterventional CT in CEA patients (who did not receive DAPT or GpIIb/IIIa inhibitors) was comparable and did not differ significantly, implies that intrainterventional hemorrhage is mediated more by reperfusion injury than antiplatelet medication. Although pathophysiological proof is missing, our findings are supported by Galyfos et al. who reported comparable to slightly elevated rates of cerebral hyperperfusion syndrome in patients receiving CEA compared to CAS [20]. The fact that maximum systolic blood pressure during the intervention did not differ significantly between patients with and without intrainterventional hemorrhage may imply that reperfusion hemorrhage occurs independently of blood pressure; however, given the small overall number of intrainterventional hemorrhages in our study, further research is needed to address whether strict blood pressure management throughout the procedure may further decrease hemorrhage rates.

Since delayed space-occupying hemorrhage (PH2) in our study occurred exclusively in CAS patients, our data suggest that early subacute parenchymal hemorrhage after CAS, however, is mainly caused by DAPT/GpIIb/IIIa inhibitors. This is supported by studies that reported increased hemorrhage rates in thrombectomy patients with CAS, as for instance, by Kellert et al. who showed that the risk of fatal ICH was significantly higher in patients receiving tirofiban after thrombectomy than in those who did not receive it (13.3% vs. 3.1%; *p* = 0.05) [21]. In their analysis of patients with either anterior or posterior occlusion, tirofiban treatment was an independent predictor for fatal ICH (OR 3.03; 95% CI 1.50–4.05; *p* = 0.04) and an independent predictor for poor outcome (OR 6.60; 95% CI 1.06–41.52; *p* = 0.04).

This is in line with our second key finding: even though overall hemorrhage rates (PH1 and PH2) were comparable in CAS and CEA patients in our study, large space-occupying hemorrhage (PH2) was more frequent in the CAS group (10/12) than in the CEA group (2/4) and there was no sICH in the CEA group, suggesting that hemorrhage in thrombectomy patients with CAS and DAPT/GpIIb/IIIa inhibitors is more likely to be space-occupying. This is supported by the results of Slawski et al. who compared their 12 CEA patients to 27 CAS patients and found no sICH in the CEA group, but 11% in the CAS group [11]. As opposed to Slawski et al. and most other studies we focused on PH rates rather than on sICH rates, because the occurrence of a hematoma has clinical implications regardless of its impact on neurological status (e.g. change of antiplatelet medication or surgery). In fact, it is quite likely that space-occupying hemorrhages in large middle cerebral artery infarctions may not result in neurological deterioration of ≥4 points on the NIHSS but nonetheless may result in modification of antiplatelet treatment or surgery. Considering this, sICH rates must be interpreted with great caution: for instance, in the TITAN registry Papanagiotou et al. reported sICH rates of 4% (5/137) vs. 7% (8/118, *p* = 0.397) in patients with tandem lesions who underwent CAS and received either one or at least two antiplatelet agents (without indicating PH rates) [22]. At first sight these numbers are lower than our indicated hemorrhage rate; however, if not only sICH but all parenchymal hemorrhages are accounted for, the actual PH rate in the TITAN registry according to Anadani et al. (in a separate analysis of 205 CAS patients of the TITAN registry) is in fact 16%, which is comparable to our data [23].

Thus, even though our study and the vast majority of studies addressing thrombectomy and CAS are limited by their small sample sizes, inhomogeneous patient cohorts with differing antiplatelet regimens (GpIIb/IIIa inhibitors, timing and dose of mono or dual antiplatelet therapy), varying hemorrhage definitions (“sICH”, “hemorrhage”, “parenchymal hemorrhage”, “intracranial hemorrhage”, etc.), and foremost a lack of randomization, elevated hemorrhage risks in thrombectomy patients with CAS (of up to 43%) support our assumption that thrombectomy and CAS with DAPT and/or GpIIb/IIIa inhibitors is associated with an increased hemorrhage rate [6–10].

Aspirin monotherapy may be an alternative to reduce hemorrhage risk after CAS, even though it has been shown that DAPT in non-thrombectomy cases is associated with superior neurological outcome.[24–26]. Although CAS with only one antiplatelet agent did not result in a significantly decrease of sICH rates in the TITAN registry (*p* = 0.397), refinement of antiplatelet therapy after CAS is an important subject for future studies [22].

Our results and data from the literature also imply that early CEA instead of CAS could be beneficial for patients. In fact, according to Reznik et al., the timing between symptom onset of acute stroke and either semi-elective CEA or CAS has decreased in the past four decades because of the more favorable outcome of early carotid treatment [27]. This is based on the results of studies stating that after stroke/symptom onset CEA (vs. CAS) and especially early CEA (vs. late) should be preferred because it is associated with a lower rate of recurrent stroke or death [28, 29].

However, with the exception of the study of Slawski et al. previous studies on direct comparisons of CEA and CAS did not deal with thrombectomy patients but with (semi-)elective patients with (a)symptomatic carotid stenosis without large-vessel occlusions that necessitate thrombectomy: Sbarigia et al. performed emergency CEA in 6 patients within the median of 6 h after symptom onset of stroke [30]. Fatal hemorrhagic transformation occurred in 1/6 (16.5%). Their selection of patients though was limited by excluding patients with impaired consciousness or infarctions larger than 2.5 cm. Azzini et al. observed no hemorrhages in 11 patients who underwent CEA 12 h after symptom onset because of a high-grade (≥70%) symptomatic carotid stenosis, 10/12 patients had a mRS at 90 days of 0–2 [31]. Our results are also supported by larger studies that compared ICH rates of CAS and CEA patients in general: In their literature review with 218,144 CEA and 18,393 CAS patients, Galyfos et al. stated that there was no difference in ICH risks between both methods [20]; however, in their population based study with 16,688 patients (14% CAS, 86% CEA), Hussain et al. agreed on the fact that ICH are rare but occur significantly more frequently in CAS than in CEA (0.85% vs. 0.42%; adjusted OR 1.77; 95% CI 1.32–2.36; *p* ≤ 0.001) [32]. Also, McDonald et al. analyzed 215,012 CEA and 13,884 CAS patients and observed that ICH risk was significantly higher after CAS in asymptomatic (0.5% vs. 0.06%; *p* ≤ 0.0001) and particularly symptomatic patients (4.4% vs. 0.8%; *p* ≤ 0.0001) [33]. Multivariate regression revealed that symptomatic patients and stenting were both independently associated with a 6–7 times higher risk for postoperative ICH. An ICH was independently associated with a 30 times increased risk of in-hospital mortality. Notably, as in the analysis of Slawski et al., the lower hemorrhage rate in our study did not affect clinical outcome, with good clinical outcome rates (mRS 0–2) in CAS and CEA patients (51% vs. 39% *p* = 0.452; Slawski et al. 70% vs. 75% *p* = 1.0) and mortality rates (22% vs. 35%, *p* = 259; Slawski et al. 18.5% vs. 0% *p* = 0.30). Bearing in mind that the decision to perform either CAS or CEA was not randomized in any of the studies, outcome results must be interpreted with great caution: clinical outcome (in our series) is influenced by a relevant selection bias, which could influence our results in either direction and does not allow reliable conclusions to be drawn. In fact, worse initial neurological condition and higher rates of comorbidities in the CEA group—even though not significant—are likely to have attributed to the non-significantly poorer outcome. Nonetheless, in consideration of our results (mainly the lower hemorrhage rates) and data from the literature, we are convinced that early CEA instead of CAS could have a positive impact on clinical outcome.

If CEA is to be established as an alternative to CAS, procedural duration is a major issue. Even though we have the privilege of having vascular surgeons who perform surgery in our angiography suite 24/7, procedural times in CEA patients are significantly longer (*p* < 0.001). This reflects the complexity of the decision-making process, recruitment of the surgical team, and the duration of the additional procedure. Even though it is likely that these delays affect clinical outcome, we are unable to specifically attribute clinical outcomes to these delays, because of our heterogeneous and biased samples without significantly different outcomes. Notwithstanding the above, further improvement of procedural times is an important aim, as delays are likely to affect clinical outcome.

## Limitations

Major limitations of our study are its relatively small sample size and its retrospective approach. Despite our relatively small cohort of CEA patients, our study showed that CEA in thrombectomy patients is safe and may therefore help in the emergency decision-making progress whenever CEA is considered as an alternative for CAS. Our retrospective approach resulted in a considerable sample bias; however, this bias would have worked against our hypothesis that hemorrhage in the CEA group was less frequent as the decision to perform CEA was not randomized but was (often) made because the interventionalists anticipated an increased hemorrhage risk. Hence, we should have expected an increased baseline hemorrhage risk in CEA patients. The fact that we observed a decreased hemorrhage rate makes it conceivable that the clinical course of CEA patients is in fact more favorable than shown in our study if CEA is performed routinely. Also, our results may not be transferrable to other hospitals, as vascular surgeons being available 24/7 as well as our angiographic suite being suitable for the CEA procedure are not common conditions.

## Conclusion

Our data suggest that parenchymal hemorrhage in CAS and CEA patients is mediated by different pathomechanisms, with reperfusion injury as a cause for intrainterventional hemorrhage and DAPT/GpIIb/IIIa inhibitor as a cause for early subacute hemorrhage. Our results also imply that emergency CEA performed in the angiography suite is a feasible alternative to CAS in thrombectomy patients and that increased hemorrhage risks in CAS patients can be anticipated by performing CEA instead of CAS whenever possible.

## References

[1] Goyal M, Menon BK, van Zwam WH, Dippel DW, Mitchell PJ, Demchuk AM (2016). Endovascular thrombectomy after large-vessel ischaemic stroke: a meta-analysis of individual patient data from five randomised trials. Lancet.

[2] Fischer U, Mono ML, Schroth G, Jung S, Mordasini P, El-Koussy M (2013). Endovascular therapy in 201 patients with acute symptomatic occlusion of the internal carotid artery. Eur J Neurol.

[3] Meyer L, Politi M, Alexandrou M, Papanagiotou P (2019). Tandem occlusions in acute ischemic stroke. Radiologe.

[4] Rodrigues M, Cunha A, Figueiredo S, Carvalho A, Veloso M, Barros P (2018). Emergent carotid artery stenting in atherosclerotic disease of the internal carotid artery with tandem intracranial occlusion. J Neurol Sci.

[5] Kim B, Kim BM, Bang OY, Baek JH, Heo JH, Nam HS (2019). Carotid artery stenting and intracranial thrombectomy for tandem cervical and intracranial artery occlusions. Neurosurgery.

[6] Cohen JE, Gomori JM, Rajz G, Itshayek E, Eichel R, Leker RR (2015). Extracranial carotid artery stenting followed by intracranial stent-based thrombectomy for acute tandem occlusive disease. J NeuroIntervent Surg.

[7] Mpotsaris A, Kabbasch C, Borggrefe J, Gontu V, Soderman M (2017). Stenting of the cervical internal carotid artery in acute stroke management: the Karolinska experience. Interv Neuroradiol.

[8] Behme D, Mpotsaris A, Zeyen P, Psychogios MN, Kowoll A, Maurer CJ (2015). Emergency stenting of the extracranial internal carotid artery in combination with anterior circulation thrombectomy in acute ischemic stroke: a retrospective multicenter study. AJNR Am J Neuroradiol.

[9] Steglich-Arnholm H, Holtmannspotter M, Kondziella D, Wagner A, Stavngaard T, Cronqvist ME (2015). Thrombectomy assisted by carotid stenting in acute ischemic stroke management: benefits and harms. J Neurol.

[10] Grigoryan M, Haussen DC, Hassan AE, Lima A, Grossberg J, Rebello LC (2016). Endovascular treatment of acute ischemic stroke due to tandem occlusions: large multicenter series and systematic review. Cerebrovasc Dis.

[11] Slawski DE, Jumaa MA, Salahuddin H, Shawver J, Humayun MJ, Russell T (2018). Emergent carotid endarterectomy versus stenting in acute stroke patients with tandem occlusion. J Vasc Surg.

[12] Wiesmann M, Kalder J, Reich A, Brockmann MA, Othman A, Greiner A (2016). Feasibility of combined surgical and endovascular carotid access for interventional treatment of ischemic stroke. J NeuroIntervent Surg.

[13] Nikoubashman O, Dekeyzer S, Riabikin A, Keulers A, Reich A, Mpotsaris A (2019). True first-pass effect. Stroke.

[14] Fiehler J, Cognard C, Gallitelli M, Jansen O, Kobayashi A, Mattle HP (2016). European recommendations on organisation of interventional care in acute stroke (EROICAS). Eur Stroke J.

[15] Hacke W, Kaste M, Fieschi C, Toni D, Lesaffre E, von Kummer R (1995). Intravenous thrombolysis with recombinant tissue plasminogen activator for acute hemispheric stroke. The European Cooperative Acute Stroke Study (ECASS). JAMA.

[16] Hacke W, Kaste M, Fieschi C, von Kummer R, Davalos A, Meier D (1998). Randomised double-blind placebo-controlled trial of thrombolytic therapy with intravenous alteplase in acute ischaemic stroke (ECASS II). Second European-Australasian Acute Stroke Study Investigators. Lancet.

[17] Pexman JH, Barber PA, Hill MD, Sevick RJ, Demchuk AM, Hudon ME (2001). Use of the Alberta Stroke Program Early CT Score (ASPECTS) for assessing CT scans in patients with acute stroke. Ajnr Am J Neuroradiol.

[18] Adams HP, Davis PH, Leira EC, Chang KC, Bendixen BH, Clarke WR (1999). Baseline NIH Stroke Scale score strongly predicts outcome after stroke: a report of the Trial of Org 10172 in Acute Stroke Treatment (TOAST). Neurology.

[19] Banks JL, Marotta CA (2007). Outcomes validity and reliability of the modified Rankin scale: implications for stroke clinical trials: a literature review and synthesis. Stroke.

[20] Galyfos G, Sianou A, Filis K (2017). Cerebral hyperperfusion syndrome and intracranial hemorrhage after carotid endarterectomy or carotid stenting: a meta-analysis. J Neurol Sci.

[21] Kellert L, Hametner C, Rohde S, Bendszus M, Hacke W, Ringleb P (2013). Endovascular stroke therapy: tirofiban is associated with risk of fatal intracerebral hemorrhage and poor outcome. Stroke.

[22] Papanagiotou P, Haussen DC, Turjman F, Labreuche J, Piotin M, Kastrup A (2018). Carotid stenting with antithrombotic agents and intracranial thrombectomy leads to the highest recanalization rate in patients with acute stroke with tandem lesions. JACC Cardiovasc Interv.

[23] Anadani M, Spiotta AM, Alawieh A, Turjman F, Piotin M, Haussen DC (2019). Emergent carotid stenting plus thrombectomy after thrombolysis in tandem strokes: analysis of the TITAN registry. Stroke.

[24] Dalainas I, Nano G, Bianchi P, Stegher S, Malacrida G, Tealdi DG (2006). Dual antiplatelet regime versus acetyl-acetic acid for carotid artery stenting. Cardiovasc Intervent Radiol.

[25] McKevitt FM, Randall MS, Cleveland TJ, Gaines PA, Tan KT, Venables GS (2005). The benefits of combined anti-platelet treatment in carotid artery stenting. Eur J Vasc Endovasc Surg.

[26] Wallocha M, Chapot R, Nordmeyer H, Fiehler J, Weber R, Stracke CP (2019). Treatment methods and early neurologic improvement after endovascular treatment of tandem occlusions in acute ischemic stroke. Front Neurol.

[27] Reznik M, Kamel H, Gialdini G, Pandya A, Navi BB, Gupta A (2017). Timing of carotid revascularization procedures after ischemic stroke. Stroke.

[28] Rantner B, Kollerits B, Roubin GS, Ringleb PA, Jansen O, Howard G (2017). Early endarterectomy carries a lower procedural risk than early stenting in patients with symptomatic stenosis of the internal carotid artery: results from 4 randomized controlled trials. Stroke.

[29] Ferrero E, Ferri M, Viazzo A, Labate C, Berardi G, Pecchio A (2014). A retrospective study on early carotid endarterectomy within 48 hours after transient ischemic attack and stroke in evolution. Ann Vasc Surg.

[30] Sbarigia E, Toni D, Speziale F, Falcou A, Sacchetti ML, Panico MA (2003). Emergency and early carotid endarterectomy in patients with acute ischemic stroke selected with a predefined protocol. A prospective pilot study. Int Angiol.

[31] Azzini C, Gentile M, De Vito A, Traina L, Sette E, Fainardi E (2016). Very early carotid endarterectomy after intravenous thrombolysis. Eur J Vasc Endovasc Surg.

[32] Hussain MA, Alali AS, Mamdani M, Tu JV, Saposnik G, Salata K (2018). Risk of intracranial hemorrhage after carotid artery stenting versus endarterectomy: a population-based study. J Neurosurg.

[33] McDonald RJ, Cloft HJ, Kallmes DF (2011). Intracranial hemorrhage is much more common after carotid stenting than after endarterectomy: evidence from the National Inpatient Sample. Stroke.

